# Can Pre-biopsy Second-Look Breast Ultrasound Affect Clinical Management? Experience From a Single Tertiary Hospital

**DOI:** 10.3389/fonc.2022.901757

**Published:** 2022-05-31

**Authors:** Li Ma, Jing Qin, Lingyan Kong, Jialin Zhao, Mengsu Xiao, Hongyan Wang, Jing Zhang, Yuxin Jiang, Jianchu Li, He Liu, Qingli Zhu

**Affiliations:** ^1^ Department of Ultrasound, Peking Union Medical College Hospital, Chinese Academy of Medical Sciences, Beijing, China; ^2^ Department of Radiology, Peking Union Medical College Hospital, Chinese Academy of Medical Sciences, Beijing, China

**Keywords:** ultrasound imaging (USG), quality improvement (QI), breast cancer, second look, unnecessary biopsies

## Abstract

**Objectives:**

Interpretation discrepancy is a major disadvantage of breast imaging. This study aimed to determine the clinical benefit of the pre-biopsy second-look breast ultrasound (US).

**Methods:**

Patients with suspicious breast masses referred to our tertiary hospital for US-guided breast biopsy were retrospectively reviewed between August 2017 and November 2019. Here, second-look assessments were performed by experienced specialized breast radiologists *via* performing a bilateral breast US scan plus reviewing former imaging studies, and results were compared with the initial assessment. Interpretation changes in terms of biopsy recommendation and surgical management (i.e., lumpectomy to mastectomy) were analyzed.

**Results:**

A total of 537 patients were enrolled in this study. Interpretation discrepancies occurred in 109 patients (20%; 95% CI, 17%–24%). Among them, there were 84 patients (16%; 95% CI, 13%–19%) whose masses were re-classified as BI-RADS 3 by the second-look US and underwent 2-year follow-up, showing 82 benign, 1 malignant, and 1 high-risk lesions. On the other hand, 16 patients (3%; 95% CI, 2%–5%) undertook biopsy at an additional site, identifying 10 new malignant lesions, 3 high-risk lesions, and 3 benign lesions, resulting in surgical management changes in 12 patients. In addition, nine (2%; 95% CI, 1%–3%) patients received discrepant disease ranges, which also altered surgical management. Overall, 21 patients (4%; 95% CI, 3%–6%) got their surgical management altered by the second-look US.

**Conclusion:**

Pre-biopsy second-look assessment of breast US can reduce unnecessary biopsies in 16% of patients and alter surgical management in 4% of patients, suggesting it is a practical and valuable method for patient care improvement.

## Introduction

Breast cancer has been the most common cancer in women worldwide for decades (1, 2). The Breast Imaging Report and Data System (BI-RADS) lexicon offers a standard strategy from imaging assessment to clinical management, making the breast imaging interpretation a critical step of patient care (3). Nevertheless, the high incidence of interpretation discrepancy is the pitfall of breast imaging, as demonstrated by previous studies (4–8). Interpretation of breast imaging requires specialized training and years of experience (9). An inaccurate diagnosis may lead to an unnecessary biopsy, impropriate surgical plan, or delayed cancer diagnosis, resulting in wasting medical expense and imposing negative influences on patients’ prognosis.

One valid measure to increase the diagnostic accuracy of breast imaging is to seek a second opinion by dedicated radiologists at specialized cancer centers. Literature has confirmed the performance difference between specialists and general radiologists (10, 11). Studies show that the second review of breast imaging causes interpretation discrepancies in 16%–57% of patients and alters changes in clinical management in 7%–27% of patients (12–15). However, as also addressed by the literature, the second consultation process is time consuming and labor intensive. The reviewing radiologist may recommend additional imaging or biopsy, which may cost extra visits and expenses to patients. Therefore, the chance of the clinical application is compromised (12). A more efficient way to optimize patient care is still awaiting.

In our tertiary medical center, a second-look assessment is routinely done prior to the biopsy step, a must-do process for all suspected breast lesions. A second-look assessment included a new ultrasound (US) scan to re-evaluate the whole breast by experienced breast specialists after full review of the other image modalities. By this, the second review is achieved with little extra labor, meanwhile, without adding more visits to patients. The goal of this retrospective study was to determine the clinical impact of the second-look assessment, including detection of new cancers, prevention of unnecessary biopsies, and changes in surgical procedures.

## Materials and Methods

### Patient Recruitment

This is a retrospective study performed in a referral tertiary hospital in China and approved by the Institutional Review Board of Peking Union Medical College Hospital. Patients referred to our breast surgery clinic between August 2017 and November 2019 were eligible for this study if fulfilling the following inclusion criteria: (1) presented with focal breast mass(s) with a BI-RADS category 4 or 5 as assessed by the initial breast imaging; (2) referred to specialized breast radiologists in our institution for US-guided biopsy; and (3) the time interval between performing initial imaging and the biopsy was within 1 month. Patients who had received breast biopsy before or between the two imaging assessments were excluded. In our institution, all US-guided biopsy candidates would receive a bilateral whole breast ultrasound re-examination during the study period. According to the sample size estimation, we randomly selected 596 patients for enrollment.

### Initial Breast Imaging Assessment and Second-Look US

The initial breast imaging includes mostly breast US and mammography, with a small percentage of MRI, which was either performed at our own clinics or at outside institutions such as community institutions, secondary hospitals, or private practices. The initial evaluations were interpreted by radiologists with varying experience and/or specialization.

The second-look breast US were performed by one of the four specialized breast radiologists who had been dedicatedly working on breast imaging performing over 3,000 breast studies per year for at least 10 years. At this time, they first performed a new, complete bilateral breast US scan using Philips iU22 or EPIQ 7 (Philips, Bothell, WA, USA), or Sumsung WS80A (Samsung Medison, Seoul, Korea) with a linear (L12–5) transducer. Based on the findings from the US scan and the review of previous breast US or mammogram images, an official radiological report was issued with detailed descriptions of any suspicious findings and BI-RADS categories.

### Comparison of the Initial and the Second-Look Evaluation

The interpretations of the initial and the second breast imaging assessment were collected and compared; the results were divided into three categories: first, biopsies not recommended by second-look assessment, i.e., a downgrade BI-RADS category change from 4/5 to category 2/3; second, biopsies recommended at additional sites by second-look assessment, i.e., an upgrade BI-RADS category change from 2/3 to category 4/5, or a new identified lesion that was undetected by the initial assessment; third, in spite of same BI-RADS categories, there were clinically significant discrepancies with disease extent, which would affect further surgical management (such as a change from lumpectomy to mastectomy). The clinical significance was judged by one breast surgeon with over 10 years of practice. Note that the third situation is only applicable to malignant masses.

### Further Management

If the second-look assessment evaluated the mass as BI-RADS 3, a follow-up with a time interval of 6 months was suggested for at least 2 years. If there were no changes in 2 years, the mass was considered as benign, or else the patient would process with further treatment.

Standard 16G core needle US-guided biopsy or US-guided surgical excision with regional anesthesia was performed if the second-look US found the mass to be suspicious irrespective of the reports of initial assessment. If a new suspected lesion was found by the second-look assessment, a biopsy would also be performed.

If there was a disparity in terms of disease extent between the initial and the second-look assessment that affected the surgical management, the opinion of the second-look assessment would be adopted by the breast surgeon.

The pathological results were collected after biopsies or further surgeries and the follow-up results. Based on pathological types, the masses were divided into three categories: benign, which included fibroadenoma, intraductal papilloma, and adenosis; malignant, which included infiltrating ductal carcinoma, infiltrating lobular carcinoma, solid papillary carcinoma, and mucinous carcinoma; and high-risk lesion, which included atypical lobular hyperplasia, atypical ductal hyperplasia, and lobular carcinoma *in situ*.

### Statistics

Statistical analysis was performed using the SPSS Statistics software (v23). Descriptive statistics were reported as number (percent) for categorical variables and mean (range) or mean ± SD for continuous variables. Percentages with 95% confidence interval (CI) were calculated.

The sample size was calculated based on the following assumption: null hypothesis, there is no significant change in the second US as compared to the initial evaluation; two-sided significance level, 0.05; power, 0.80; the difference between the second the initial evaluation, 5%. This resulted in at least 473 participants needed for our study.

## Results

### Patient Information

In the study period, 596 patients were eligible for our study. Fifty-nine patients who already performed biopsy before the second-look assessment were excluded, resulting in a final enrollment of 537 patients. There included 5 (1%) male patients and 532 (99%) female patients; the median age was 45 (ranged from 12 to 91). Breast cancer history was found in 31 (6%) patients. Most patients (474, 88%) took both mammography and US studies, while 13 (2%) patients also took MRI examinations. Fifty (9%) patients only took US studies. According to the final pathological plus follow-up results, 267 (50%) patients received a benign diagnosis, 230 (43%) received malignant diagnosis, and 40 (7%) were diagnosed with high-risk lesions. Fibroadenoma (70, 13%) and infiltrating ductal carcinoma (189, 35%) were the most common pathological types. The basic information is listed in detail in **Table 1**.

**Table 1:** Basic information of enrolled patients.

Characteristics	Number (n = 537)
**Patients**	756:5
Age, median (range), years	45 (12-91)
Menopause, n (%)	214 (40)
Gestation/lactation, n (%)	2 (0.4)
Breast cancer history, n (%)	31 (6)
Breast cancer history of first relatives, n (%)	32 (6)
**Breast imaging modalities, n (%)**	
US only	50 (9)
US and mammography	474 (88)
US, mammography, and MRI	13 (2)
**Final pathological or follow-up results, n (%)**	
Benign	267 (50)
Fibroadenoma	70 (13)
Intraductal papilloma	56 (10)
Adenosis	38 (7)
Other benign types^1^	21 (4)
Followed up benign	82 (15)
Malignant	230 (43)
Infiltrating ductal carcinoma	189 (35)
Other malignant types^2^	41 (8)
High-risk lesions	40 (7)

^1^Other benign types include normal breast tissue, ductal dilation, infection, benign phyllodes tumor, fat necrosis, cyst, hamartoma, mesenchymal tumor, fibromatosis, tubular adenoma, and nipple adenoma.

^2^Other malignant types include infiltrating lobular carcinoma, solid papillary carcinoma, malignant phyllodes tumor, apocrine carcinoma, infiltrating micropapillary carcinoma, mucinous carcinoma, metastatic breast cancer, encapsulated papillary carcinoma, infiltrating papillary carcinoma, malignant phyllodes tumor, lymphoma, tubular carcinoma, neuroendocrine carcinoma, tall cell variant of papillary breast carcinoma, metaplastic carcinoma, and Paget’s disease.

### Overview of Interpretation Discrepancies

**Figure 1:**
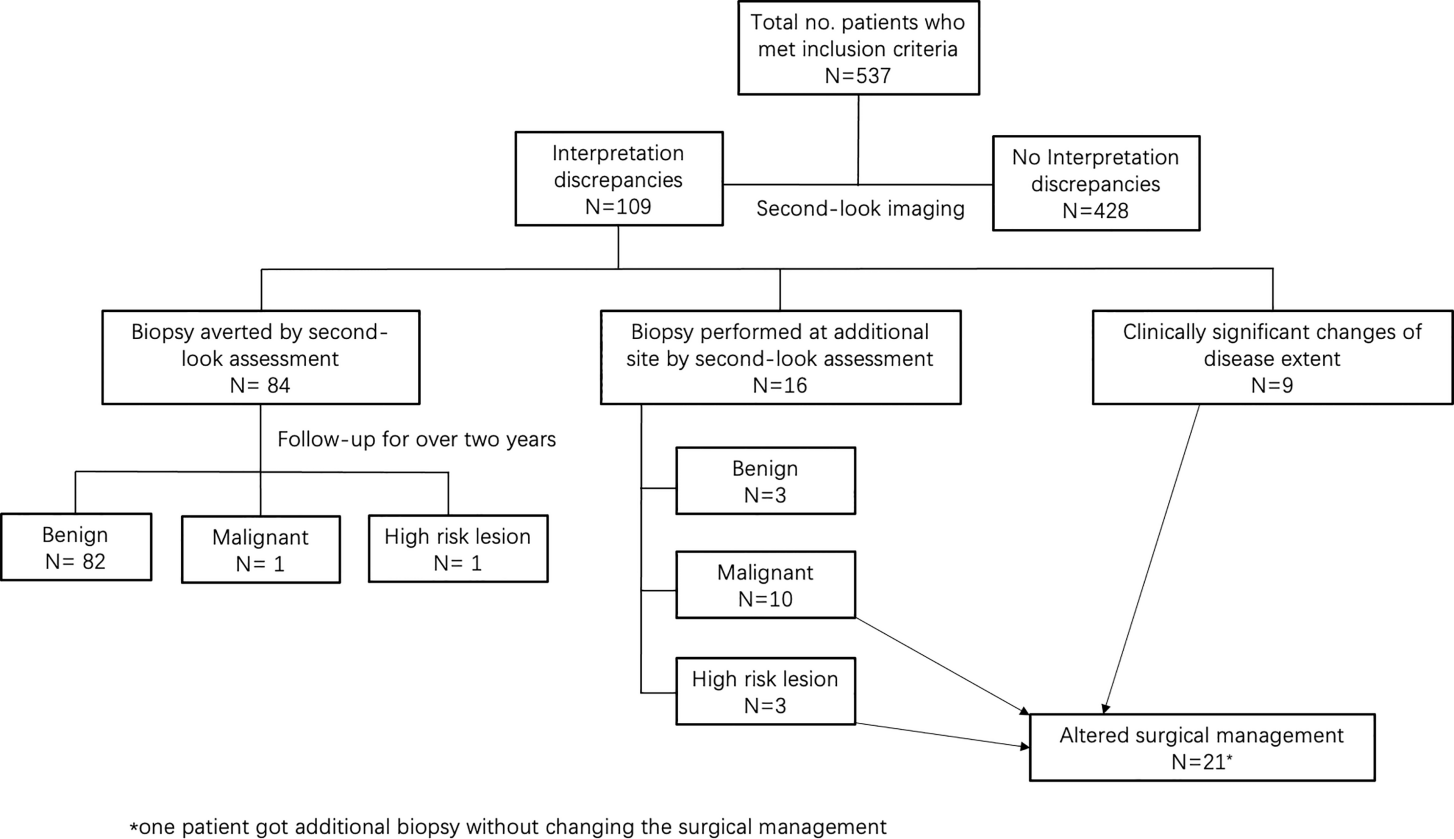
Diagram presents results of pre-biopsy second-look assessment.

Interpretation discrepancies occurred to 109 (20%; 95% CI, 17%–24%) patients (**Figure 1**). Of them, changes in the BI-RADS categories occurred in 100 patients, including 77% (84/109) downgrade changes and 15% (16/109) upgrade changes. In other 8% (9/109) patients, the extent of the disease changed without BI-RADS categories change.

### Downgrade Changes Made by Second-Look US

Of the 84 (16%; 95% CI, 13%–19%) patients whose biopsies were not recommended by the second-look assessment, most BI-RADS changes were from 4a to 3 (73%, 61/84) (**Table 2**). After a 2-year follow-up, 82 of the downgrade lesions were unchanged or disappeared, resulting in a benign diagnosis, while lesions of two patients grew bigger and finally received surgery, with one solid papillary carcinoma (largest diameter 0.5cm) and one lobular carcinoma *in situ* (largest diameter 0.2 cm). Overall, the accuracy of downgrading benign lesions by second-look assessment is 97.6%.

**Table 2:** Details of lesions with interpretation changes.

	Categories	patient number (total = 109)	Recommendations after second-look ultrasound
**Downgrade changes in BI-RADS categories**	4a→3	61	6-month follow-up
	4b→3	22	
	4c→3	1	
**Upgrade changes in BI-RADS categories, or newly founded lesions**	3→4a	2	Additional biopsy
	3→4b	4	
	3→4c	2	
	Newly identified 4a	1	
	Newly identified 4b	1	
	Newly identified 4c	2	
	Newly identified 5	4	
**Clinically significant changes of disease extent**	/	9	Surgical management changes

### Upgrade Changes or Newly Found Lesions Made by Second-Look US

**Figure 2:**
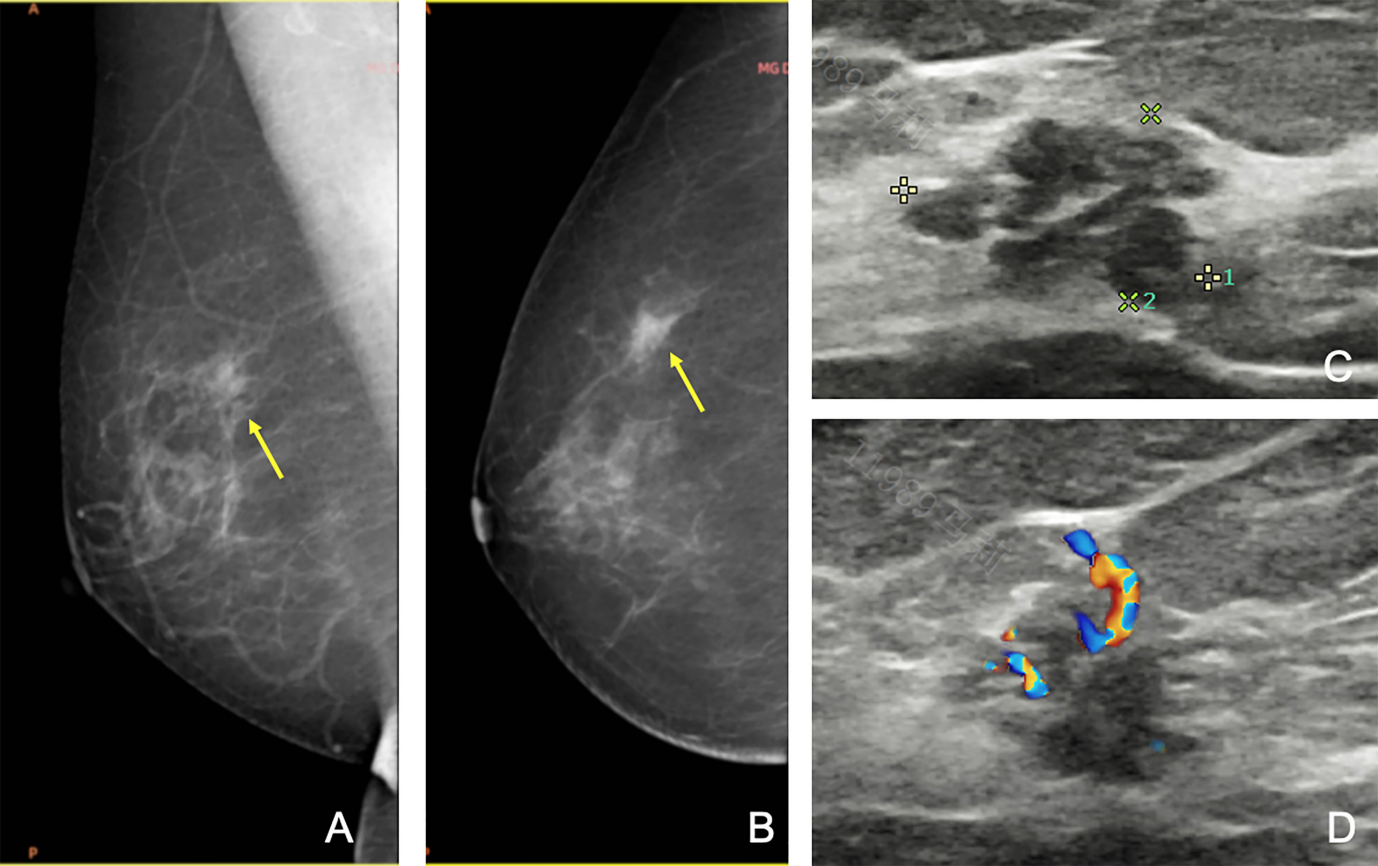
A 69-year-old woman was referred to our hospital for a category 5 mass on the left breast, and the second-look US found a new 4a mass on the right breast, which was missed at the initial assessment. Craniocaudal **(A)** and mediolateral oblique **(B)** mammograms of the right breast showing focal asymmetric dense tissue (arrows). However, since the first ultrasound failed to find any lesions, this lesion was unreported. **(C, D)** Second US showing a heterogenous lesion with micro-lobulated margin and linear vascularity. After reviewing the mammography, this lesion was re-categorized as 4a. Subsequent biopsy confirmed ductal *in situ* carcinoma. The patient finally received breast conservative surgery on both sides.

**Figure 3:**
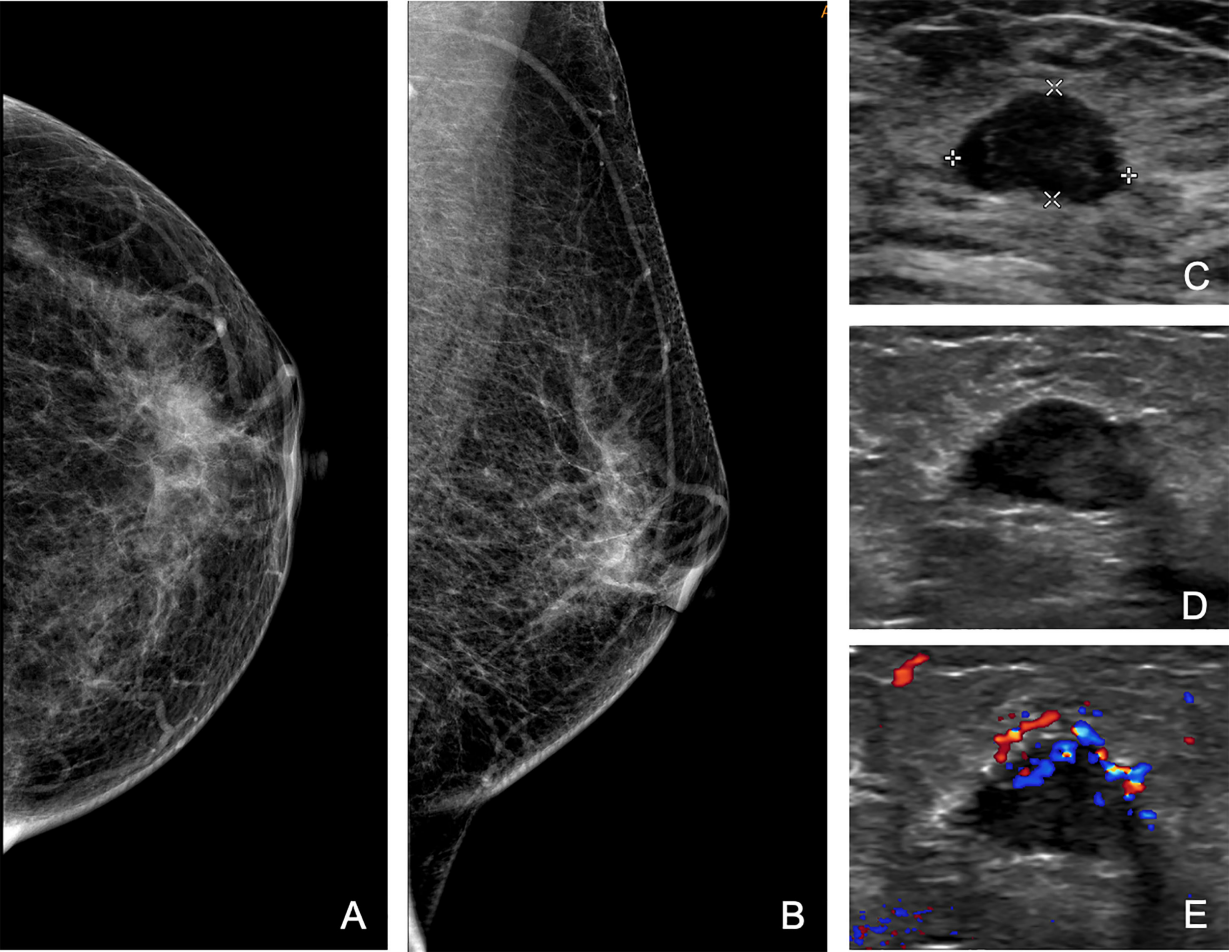
A 65-year-old woman was referred to our hospital for a category 4b mass on the right breast, and the second US found a new 4b mass on the left breast, which was categorized as 3 at the initial assessment. Craniocaudal **(A)** and mediolateral oblique **(B)** mammograms of left breast, and initial US **(C)** of the left mass. **(D, E)** Second US showing a hypoechoic mass with micro-lobulated margin and enriched vascularity on the axial view. Subsequent biopsy revealed infiltrative ductal carcinoma.

Additional biopsies were performed in 16 (3%; 95% CI, 2%–5%) patients. Eight additional biopsies were newly found masses, and eight were formerly BI-RADS 3 lesions (**Table 2**). The pathological analysis identified 10 new malignant masses, 3 high-risk masses, and 3 benign masses. The positive predictive value of additional biopsies was 81% (13/16). Eight out of 10 were additional ipsilateral malignancies. In the other two patients, new malignant masses were detected on the contralateral breast, with a missed ductal carcinoma *in situ* and an infiltrative ductal carcinoma originally categorized as BI-RADS 3 (**Figures 2**, **3**). The three high-risk lesions were all newly identified masses, including two lobular carcinomas *in situ* and one atypical lobular hyperplasia. The original suspected mass was pathologically benign in one patient and malignant in two patients. Additional lumpectomy was subsequently performed.

### Changes Leading to Alteration in the Surgical Management

Surgical management was altered in 21 (4%; 95% CI, 3%–6%) patients (**Table 3**). This included 12 patients who received additional biopsies with new malignancies or high-risk lesions found, as stated above. One patient was excluded because she intended to undertake mastectomy with the initial assessment owing to the tumor size >5 cm, and a second-look assessment performed an additional biopsy and confirmed a new ipsilateral malignancy in another quadrant, but her surgical plan was not changed. Of the 12 patients, 7 patients with new ipsilateral malignancies got their surgical plans changed from breast conservative surgery to mastectomy; 2 patients with new contralateral malignancies received either additional contralateral breast conservative surgery or mastectomy on the contralateral side. For the three patients with newly found high-risk lesions, all of them received additional lumpectomy.

**Table 3:** Patients with altered surgical management by the second-look US.

Categories	Total patients = 21	Descriptions
New ipsilateral malignancies	7	Focal IDC to multicentric IDC; surgical management changed from BCS to mastectomy
New contralateral malignancies	2	One patient received additional contralateral BCS; one patient took mastectomy on the contralateral side
New high-risk lesions	3	One ALH and two LCIS; received additional lumpectomy
Significant extent change	9	Surgical management changed from BCS to mastectomy

**Figure 4:**
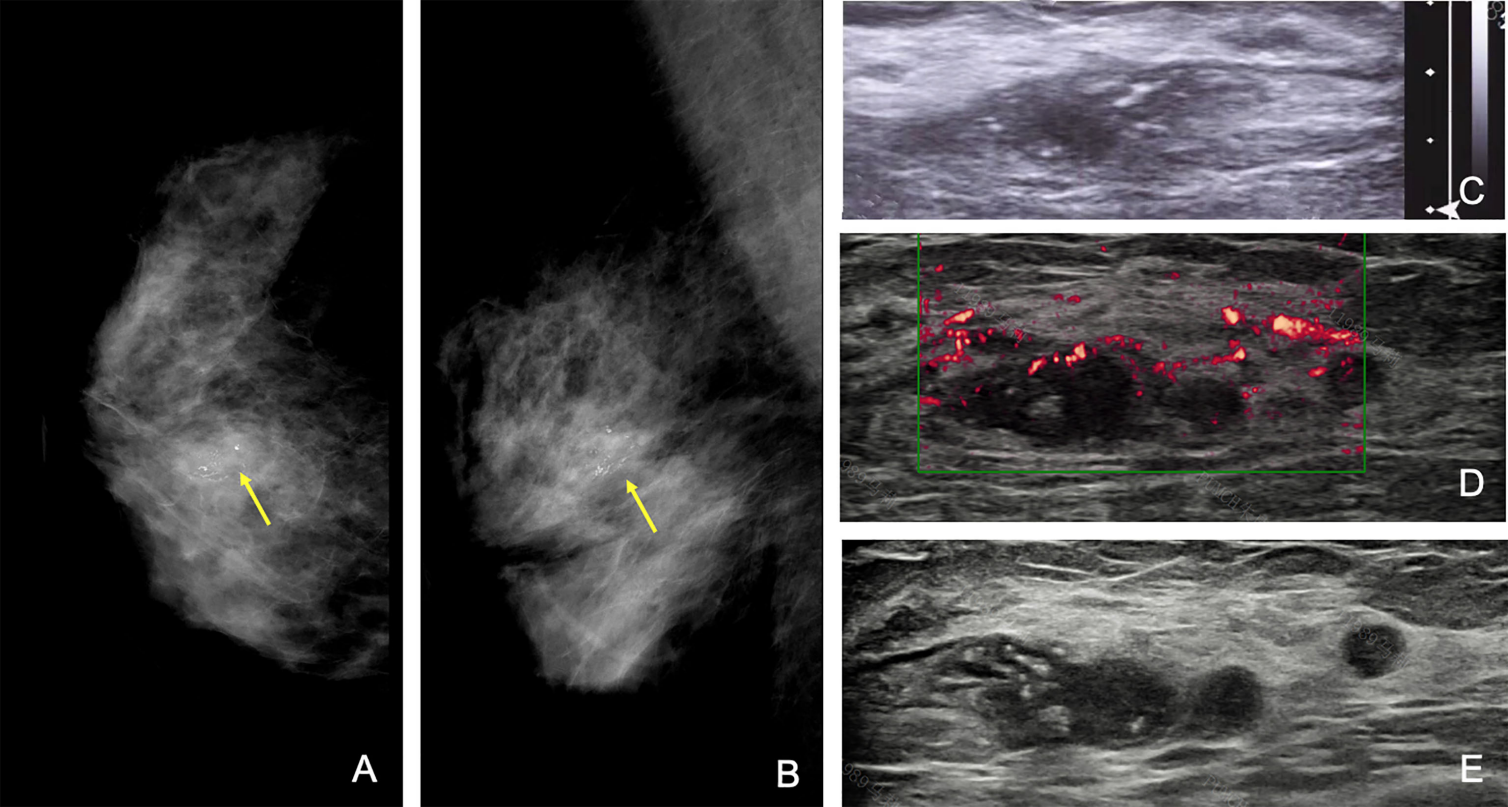
A 58-year-old woman with a category 4c mass with grouped fine branched calcification at 1 o’clock of the right breast, with the largest diameter of 1.5 cm at the initial assessment. Craniocaudal **(A)** and mediolateral oblique **(B)** mammograms of the right breast and initial US **(C)** of the right mass (arrow). The initial largest diameter was assessed as 1.8 cm. **(D)** Second US showing an irregular hypoechoic mass with multiple satellite nodules (arrows), and the largest diameter was 4.3 cm. The patient finally received mastectomy of the right breast, and pathological results were infiltrative ductal carcinoma and ductal carcinoma *in situ*, with a total range of approximately 4.0 cm. Note that the time interval between the first and second US studies was 2 days.

Besides the above situations, there were 9 (2%; 95% CI, 1%–3%) patients receiving discrepant descriptions of breast disease, which had a significant impact on surgical management. For example, one 58-year-old patient presented with right breast fine linear branched calcification on mammography and one irregular hypoechoic lesion on US, with a maximum diameter of 1.8 cm by the initial assessment. The second-look US revealed a blurred hypoechoic mass with multiple satellite nodules and measured the largest diameter of 4.3 cm. Finally, this patient underwent a mastectomy, and pathological analysis proved infiltrative ductal carcinoma and ductal carcinoma *in situ*, with a total range of approximately 4.0 cm (**Figure 4**).

## Discussion

In this study, we set a second-look breast US assessment performed by specialized breast radiologists in a high-level tertiary hospital before the US-guided biopsy and find that this measure can change clinical management up to 20% of patients, including 16% of patients being restrained from biopsies and 4% of patients getting changed in surgical management. Our results verify that second-look radiological assessment is a plausible method to maintain high diagnostic accuracy.

In Asia, US is a more popular breast imaging modality than mammography, as dense and small breasts are more common in the Asian population, which compromises the performance of mammography (16). Nevertheless, the high operator dependence is a main disadvantage of US, even after decades of application of BI-RADS lexicon (7, 17–20). In a previous study, more experienced radiologists tend to have higher consistency and better diagnostic performance (10). A second look by specialized radiologists is believed to increase the diagnostic performance. Several studies have applied second opinion reviews to patients with or without breast cancer diagnosis in dedicated cancer centers. The reviews were conducted by specialized radiologists with a re-interpretation of radiological, histological, clinical, and demographic data. Only in one study by Weinfurtner et al., axillary US was performed in patients with over 2-cm suspected invasive breast cancer; otherwise, no new examinations were performed (15). Previous studies showed rates of discrepancy between 16% and 49% and rates of change in the surgical management between 6% and 27% (12–14, 21, 22). Our study included patients with suspected breast cancer who had not yet undertaken biopsies. In our reports, the rates of discrepancy and rate of change in the surgical management are 21% and 4%, respectively, close to earlier reports. An advantage of our design is that it is time and labor saving compared to previous studies, as we combined the visit of re-consultation and biopsy, and when new suspicious lesions were found, immediate biopsies would be done without another appointment. Collectively, we got similar diagnostic improvements by exerting fewer extra efforts.

In our study, in addition to reviewing initial images and reports, specialized breast radiologists were also required to perform a repeated whole-breast US scan and gave their second opinion based on both initial and new images. This is based on the thought that image acquisition and interpretation are both important. For example, **Figure 3** presents the hidden features of differentiating 3 from 4a; thus, the error is at the level of US image interpretation. **Figure 4**, on the other hand, implies the incomplete cross-section of a malignant mass; thus, the error is at the level of US image acquisition.

Most interpretation discrepancies occur to lesions that were recommended for biopsy initially but got refuted by the second-look US. Most changes are category 4a to 3, indicating that this is a focused problem, especially for inexperienced radiologists who cannot master the fine distinction between lobulated and micro-lobulated shapes, slightly indistinct margins, or suspected signs of calcifications (23). We report that 97.6% of downgrade lesions were benign, at the expense of missing one malignancy and one high-risk lesion, proving that second-look US can effectively reduce unnecessary biopsies.

Based on our results, the positive prediction rate of additional biopsies is 81.3% (13/16). In the other two similar studies, the positive prediction rates of additional biopsies are also as high as 54.3% (50/92) and 70% (14/20) (12, 15). This shows the significance of the second-look opinions. This result demonstrates the value of second-look assessment by detecting more malignancies without causing a high rate of false-positive biopsies.

Other measures aiming to improve the interpretation discrepancy are also recorded. New imaging techniques are already playing a role in breast cancer diagnosis. For example, elastography has been recommended by the European Federation of Societies for Ultrasound in Medicine and Biology (EFSUMB) guidelines for increasing diagnostic confidence, which will decrease operator dependence by providing more information (24). Nowadays, new computer-aided detection technology, especially with artificial intelligence, has shown promising potential to improve diagnostic consistency (25). Further exploration is needed to validate these new techniques.

There are several limitations to our study. First, the study is performed in a single center, although we believe that our institution represents a general situation of the diagnostic performance of tertiary breast cancer centers. Second, we only focus on patients who are suitable for US-guided biopsy; thereby, our findings may not be applicable for US-negative breast cancer. Third, new techniques, such as shear wave elastography, have been proven helpful in increasing the diagnostic performance by high-quality studies (26). Unfortunately, due to the SWE data not being routinely available in the breast US, we were not able to analyze the SWE additional value to the second-look US in the retrospective study. Future studies can be designed to address this topic.

In conclusion, pre-biopsy second-look breast US can greatly reduce unnecessary biopsies and alter surgical management, confirming it as a practical and valuable method for patient care improvement.

## Data Availability Statement

The original contributions presented in the study are included in the article/supplementary material. Further inquiries can be directed to the corresponding authors.

## Ethics Statement

The studies involving human participants were reviewed and approved by The Institutional Review Board of Peking Union Medical College Hospital. Written informed consent to participate in this study was provided by the participants’ legal guardian/next of kin.

## Author Contributions

LM, QZ, and HL designed the study. LM was in charge of data collection, IRB review, informed consent, and manuscript daft writing. JQ collected all information from patients and doctors and performed part of data analysis. LK, MX, HW, and JinZ performed the second-look ultrasound and reviewed initial assessment. JiaZ performed the statistical analysis. YJ, JL, and ZQ made manuscript revision and supervised the study. All authors contributed to the article and approved the submitted version.

## Funding

This study was funded by the CAMS Innovation Fund for Medical Sciences (grant number 2020-I2M-C&T-B-033) and National Natural Sciences Foundation of China (grant number 81971640).

## Conflict of Interest

The authors declare that the research was conducted in the absence of any commercial or financial relationships that could be construed as a potential conflict of interest.

## Publisher’s Note

All claims expressed in this article are solely those of the authors and do not necessarily represent those of their affiliated organizations, or those of the publisher, the editors and the reviewers. Any product that may be evaluated in this article, or claim that may be made by its manufacturer, is not guaranteed or endorsed by the publisher.
